# Potential clinical impact of metagenomic next-generation sequencing of plasma for cervical spine injury with sepsis in intensive care unit: A retrospective study

**DOI:** 10.3389/fcimb.2022.948602

**Published:** 2022-08-09

**Authors:** Jian Wan, Liwei Duan, Qitong Chen, Lv Wang, Jinxia Bai, Jingyun Hu, Xinyuan Lu, Tao Zhang, Wei Song, Degang Yang, Yi Shan, Zhu Yan

**Affiliations:** ^1^ Department of Emergency and Critical Care Medicine, Shanghai Pudong New Area People’s Hospital, Shanghai, China; ^2^ Department of Emergency and Critical Care Medicine, Shanghai Changzheng Hospital, Shanghai, China; ^3^ Central Lab, Shanghai Key Laboratory of Pathogenic Fungi Medical Testing, Shanghai Pudong New Area People’s Hospital, Shanghai, China; ^4^ Department of Infectious Diseases, Shanghai Skin Disease Hospital, Tongji University School of Medicine, Shanghai, China

**Keywords:** cervical spine injury, metagenomic next-generation sequencing, sepsis, ICU, a retrospective study

## Abstract

Cervical spine injury (CSI) accounts for significant mortality in the intensive care unit (ICU), whereas sepsis remains one of the major causes of death in patients with CSI. However, there is no effective method to diagnose sepsis timely. The aim of this study is to investigate the effect of metagenomic next-generation sequencing (mNGS) on the pathogen features and the prognostic prediction of CSI patients with sepsis. A total of 27 blood samples from 17 included patients were tested by mNGS. Data of mNGS were compared with the conventional culture method. The Kaplan–Meier plots were used to visualize survival curves. A Cox proportional hazards model was used to identify independent prognostic factors for survival. Results showed that mNGS detected a wide spectrum of pathogens in CSI patients with sepsis, including 129 bacterial species, 8 viral species, and 51 fungal species. mNGS indicated 85.2% positive results, while the conventional culture method only showed 11.1% positive results in the blood samples. Further analyses revealed that mNGS had no prognostic effect on the septic CSI patients in ICU, whereas positive results of blood culture were closely correlated with an increased hazard ratio (HR) (HR 77.7067, 95%CI 2.860–2641.4595, *p* = 0.0155). Our results suggested that the mNGS application may provide evidence for clinicians to use antibiotics when a CSI case is diagnosed with sepsis.

## Introduction

Sepsis is a life-threatening organ dysfunction resulting from dysregulated host response to infection (Singer et al., 2016; Cecconi et al., 2018). In 2017, an estimated 48.9 million cases of sepsis were recorded worldwide, and 11.0 million sepsis-related deaths were reported globally (Rudd et al., 2020). In China, the standardized sepsis-related mortality rate is high, accounting for 66.7 deaths per 100,000 people (Weng et al., 2018). Multiple studies have shown that sepsis is the main cause of intensive care unit (ICU) admission and mortality (Engel et al., 2007; Fleischmann et al., 2016). However, there is no approved specific molecular therapy for sepsis. Timely identification, targeted treatment, and supportive care are necessary to improve clinical outcomes. Sepsis can virtually originate from any infecting pathogens, including bacteria, viruses, fungi, and parasites. Blood culture has been considered the gold standard for sepsis diagnosis. However, microbiological confirmation is difficult to complete when treatment starts. Even when microbiological tests are completed, culture-positive sepsis is observed in only 30%–40% of all cases (Singer et al., 2016). Furthermore, conventional blood culture takes 2–3 days to obtain initial results and up to 1 week for confirmation, resulting in delayed or inadequate targeted antimicrobial therapy (Gu et al., 2019; Han et al., 2019). Alternatively, polymerase chain reaction (PCR)-based techniques have been introduced for pathogen detection in the past decades. However, PCR-based techniques are limited by targeting few numbers of pathogens using specific primers or probes, and a very small portion of the genome (Chiu and Miller, 2019; Gu et al., 2019), and there is also a constant need to update PCR-based methods to include emerging resistance genes and mutations (Buchan and Ledeboer, 2014). Thus, a more sensitive, rapid, and accurate approach for identifying infections of sepsis is needed.

Metagenomic next-generation sequencing (mNGS) is a novel and culture-independent high-throughput sequencing method for evaluating infection and features the advantage of shortened turnaround time, unbiased detection, and semi-quantitative value in follow-up (Gu et al., 2019; Han et al., 2019). The capacity to detect all potential pathogens in a sample and simultaneously interrogate host responses makes it a potential tool in the diagnosis of infectious diseases. To date, several studies have provided a glimpse into the value of mNGS in finding out the causative pathogens and guiding targeted antimicrobial therapy. In 2014, Wilson et al. reported the first use of clinical mNGS for actionable diagnosis and treatment in a critically ill patient with a mysterious neurological infection, as the successful diagnosis prompted appropriate targeted antibiotic treatment and eventual recovery of the patient (Wilson et al., 2014). Increasing data provided by mNGS has been leveraged for detecting pathogens in central nervous system infections, bloodstream infections, respiratory infections, gastrointestinal infections, and ocular infections (Gu et al., 2019). Therefore, mNGS can be a key driver for the precise diagnosis of infectious diseases.

Cervical spine injury (CSI) is defined as the damage to the cervical spinal cord, with traumatic and non-traumatic etiologies. It is associated with significant morbidity and mortality in patients in ICU (Ahuja et al., 2017). The incidence of CSI for the general population is 0.13 per 1,000 (Wu et al., 2012), and mortality in patients with CSI has been reported to be three times more than that in age-matched healthy subjects (Ghajarzadeh et al., 2019). CSI leads to an increased rate of sepsis, which remains to be one of the main causes of death following CSI (Cao et al., 2019; Buzzell et al., 2020; Kamp et al., 2020). Thus, it is necessary to timely diagnose sepsis and deliver the therapeutic actions targeting sepsis to the patients with CSI.

Here we performed a retrospective study to evaluate the potential clinical impact of mNGS on plasma for CSI patients with sepsis in ICU and to investigate the effect of mNGS on the prognosis of these patients. We found a wide spectrum of pathogens in CSI patients with sepsis, including 129 bacterial species, 8 viral species, and 51 fungal species. mNGS indicated 85.2% positive results, while the conventional culture method only showed 11.1% positive results in the blood samples. Further analyses revealed that positive results of blood culture were closely correlated with an increased hazard ratio (HR).

## Methods

### Study population and specimen collection

The Ethical Review Committee of Changzheng Hospital approved this study. Written informed consent was received from participants or their legal guardians prior to inclusion in this study. All patients in the ICU at Changzheng Hospital from February 2018 to June 2019 were enrolled in this study. The inclusion criteria were 1) patients with complete medical records and with a temperature above 38°C or below 36°C, 2) accompanied with mNGS results for blood samples, and 3) diagnosed with CSI. 4) Patients should also meet one of the following conditions: a) with definite invasive sites or migration foci, b) with rashes or bleeding spots with unknown cause or an increasing blood neutrophil level, and c) systolic pressure below 90 mmHg or decreasing more than 40 mmHg. The exclusion criteria were patients diagnosed with a malignant tumor, blood-related diseases, chronic viral infection, severe major organ dysfunction, or chronic inflammatory diseases.

### Patient demographics and clinical features

The demographic data reviewed were based on the information provided by the patients at the time of admission into ICU, including age and gender. Clinic data observed during the first 24 h of the hospital stay were collected to obtain the following variables: diagnosis, temperature (°C), heart rate, respiratory rate, systolic and mean arterial blood pressure (mmHg), PaO_2_ or FiO_2_ (mmHg), arterial pH and bicarbonate, serum sodium, potassium, urea and creatinine, urine output, white blood cell count, hematocrit, platelet count and bilirubin, Acute Physiology and Chronic Health Evaluation II (APACHE II) score (Knaus et al., 1985), and sequential [sepsis-related] organ failure assessment (SOFA) score (Vincent et al., 1996). For the patients with multiple blood samples, APACHE II and SOFA scores were timely evaluated when the blood sample was acquired.

### Sample preparation and processing

A venous blood sample at a volume of 5 ml was collected in Streck cell-free DNA (cfDNA) tubes and stored at 4°C before use. Samples were collected at the time of suspected or confirmed sepsis diagnosis. The tubes were spun down at 1,600 *g* for 10 min at room temperature to prepare plasma. cfDNA was extracted from plasma using QIAamp (QIAGEN, Valencia, CA, USA) following the manufacturer’s operational manual. The extracted DNA was used for the construction of DNA libraries.

### Sequencing

Sequencing mNGS libraries were prepared. Negative controls and positive controls were included with patient samples in each batch. The quality of the DNA libraries was evaluated by Agilent 2100 Bioanalyzer (Agilent Technologies, Santa Clara, CA, USA). High-throughput sequencing was conducted using the Illumina NextSeq550Dx platform. On average, twenty-five million reads from the sample were acquired on the sequencer.

### Reference database and quality control

Reference genomes for *Homo sapiens* and microorganisms (bacteria, viruses, fungi/molds, and other eukaryotic pathogens) were retrieved from the National Center for Biotechnology Information (NCBI) ftp site (NCBI, U.S. National Library of Medicine (NLM); Human Genome, release GRCh38.p7; and NCBI, U.S. NLM, Microbial Genomes, respectively). Sequence similarities between microorganism references were inspected to identify taxonomic mislabeling and sequence contamination. From the reference genomes passing these quality controls (QCs), a subset was selected to maximize sequence diversity. As part of the selection process, NCBI BioSample data were used to ensure the inclusion of reference genomes from both clinical and non-clinical isolates. Selected sequences were collected into a single FASTA file and used to generate our microorganism reference database.

The selection of MedcareDx’s proprietary microorganism reference database was performed as follows. A candidate list was generated by two board-certified infectious disease physicians by including a) DNA viruses, culturable bacteria, additional fastidious and unculturable bacteria, mycobacteria, and eukaryotic pathogens from a number of infectious disease references; b) organisms in the pathogen database referenced in published case reports; c) reference genomes sequenced from human clinical isolates (as indicated by the NCBI BioSample resource) with publications supporting pathogenicity. Organisms from the above list that were associated with high-quality reference genomes, as determined by our reference database QC process (see above), were used to further narrow the range. Finally, organisms observed as sporadic environmental contamination were excluded from the clinical reportable range (CRR) in order to prevent false-positive calls, e.g., *Propionibacterium acnes*, *Acinetobacter lwoffii*, and several *Methylobacterium* spp. The sequence database is continuously curated to minimize human cross-reactivity as well as cross-reactivity between pathogens and is screened to mitigate contamination with sequences from humans or other organisms.

### Preprocessing

Raw sequencing output files were processed to generate the demultiplexed sequencing reads files. Reads were filtered based on sequencing quality and trimmed based on partial or full adapter sequence. Low-quality base calls and adapter sequences were trimmed off from the raw reads, retaining reads of trimmed length ≥35 bp, and duplicates were removed. The Burrows-Wheeler Aligner (BWA) tool was used to align the remaining high-quality sequencing reads against the hg19 human reference sequence. Sequencing reads exhibiting strong alignment against the human references were collected, and reads identified as human were excluded from further analysis. The remaining reads were aligned against MedcareDx’s proprietary microorganism reference database.

### Determination of pathogens

To identify the pathogenic sequences, the remaining unmapped sequences were aligned to a curated pathogen database, including bacterial, viral, fungal, and parasite. The quantity for each organism identified was expressed in normalized number (NN) of DNA sequencing reads from the reported organism present in plasma. An organism in a patient was removed, as the normalized number of strictly mapped DNA sequencing reads was less than 3. For each discrete viral or bacterial genus, assigned NGS reads are directly mapped to all nucleotide reference sequences corresponding to that genus at the species, strain, or substrain level using BLASTn at an E-value cutoff of 1,020. For each genus, a coverage map of the reference sequence with the highest percent coverage is generated, with priority given to reference sequences in the following order: 1) complete genomes, 2) complete sequences, or 3) partial sequences/individual genes. The parameters for bacteria, viruses, and fungi were annotated with all classified mapped reads. A mixture model was used to assign a likelihood to the complete collection of sequencing reads in the sample. An expectation–maximization algorithm was applied to compute the maximum likelihood estimate of each taxon abundance. Only taxa whose abundances rejected the null hypothesis of originating from environmental contamination (as calculated from the negative controls) at high significance levels were reported. Typically, it took less than 29 h to complete the entire process from DNA extraction to analysis.

### Criteria for a positive metagenomic next-generation sequencing result

In this study, positive data were considered as the mapping read number, or relative abundance was more than zero. When categorizing the frequency of pathogens in the blood sample, the positive relative abundance of pathogens in each blood sample was calculated.

### Statistical analysis

All statistical analyses were performed using SPSS Statistics (version 25.0) and R (version 3.6.2) with the following main packages: forestplot, survival, survminer, and ggplot2. Continuous data were presented as mean and standard deviation. Categorical data were compared using chi-squared tests. The Kaplan–Meier plots were used to visualize survival curves and compared using the log-rank test. A Cox proportional hazards model was used to identify independent prognostic factors for survival. In stratum-specific analyses of mNGS results, the results of bacteria, viruses, and fungi were counted separately. A *p*-value of less than 0.05 was considered statistically significant.

## Results

### Demographic features

**Figure 1:**
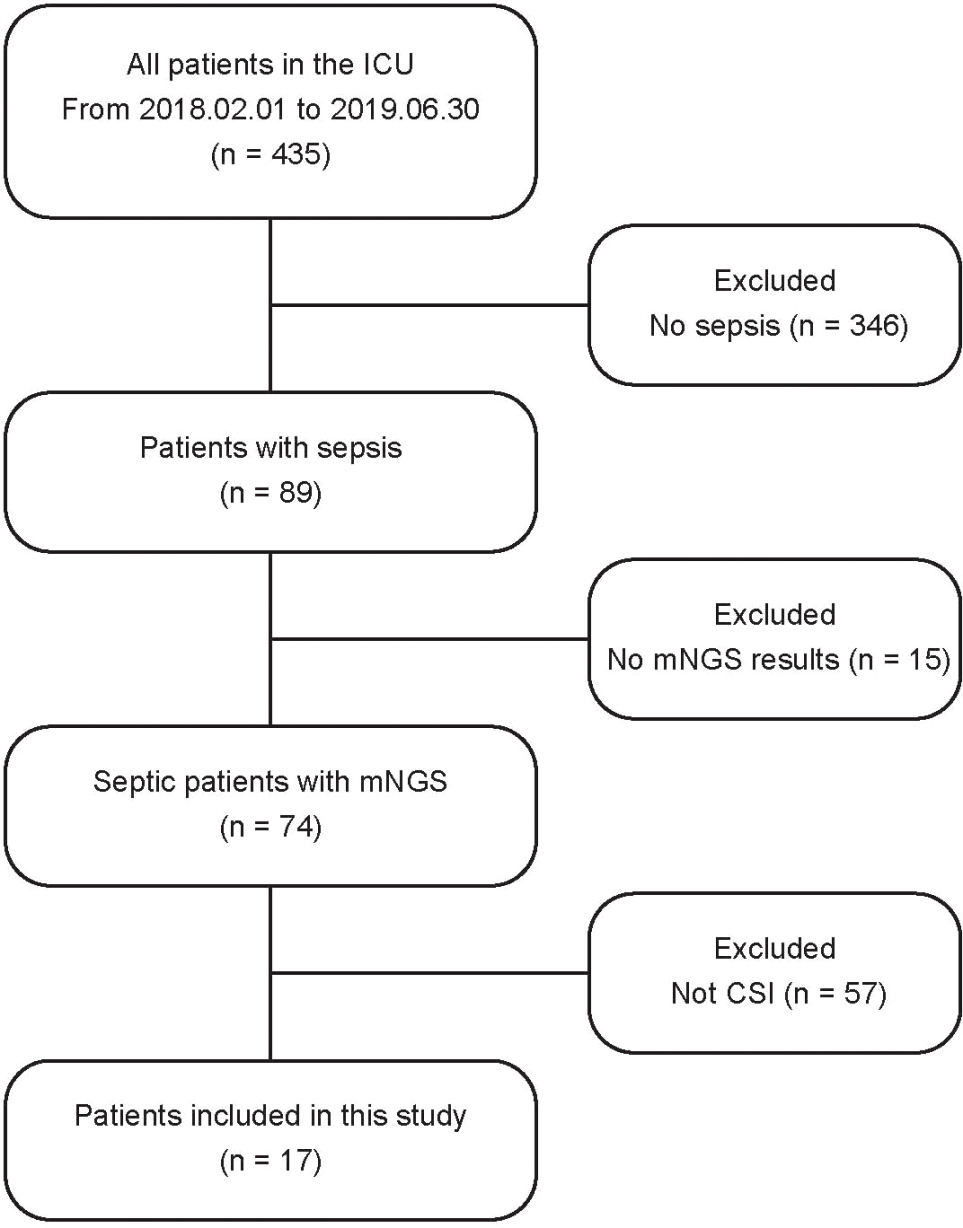
Flowchart diagram. The recruiting process of patients in the study.

From February 2018 to June 2019, a total of 435 patients were admitted to the ICU at Changzheng Hospital, and finally, 17 CSI patients with sepsis were enrolled in this study (**Figure 1**). Patients’ demographics and clinical features are described in **Table 1**. The median age was 60 (33–74) years, and 76.5% of cases were male. A total of 15/17 (88.2%) cases were traumatic CSI, and the rest two cases were non-traumatic CSI. The median APACHE II score and SOFA score were 19 (8–23) and 6 (3–12), respectively. The length of stay (LOS) in ICU ranged from 6 to 39 days.

**Table 1:** Demographics and clinical characteristics of 17 patients enrolled.

Characteristics	
Median age (range) (years)	60 (33–74)
Gender	
Male	13 (76.5%)
Female	4 (23.5%)
Etiologies	
Traumatic	15 (88.24%)
Non-traumatic	2 (11.76%)
Median APACHE II score (range)	19 (8–23)
Median SOFA score (range)	6 (3–12)
Median LOS (range) (day)	19 (6–39)

APACHE, Acute Physiology and Chronic Health Evaluation; SOFA, sequential [sepsis-related] organ failure assessment; LOS, length of stay.

### Metagenomic information of pathogens in the plasma

**Figure 2:**
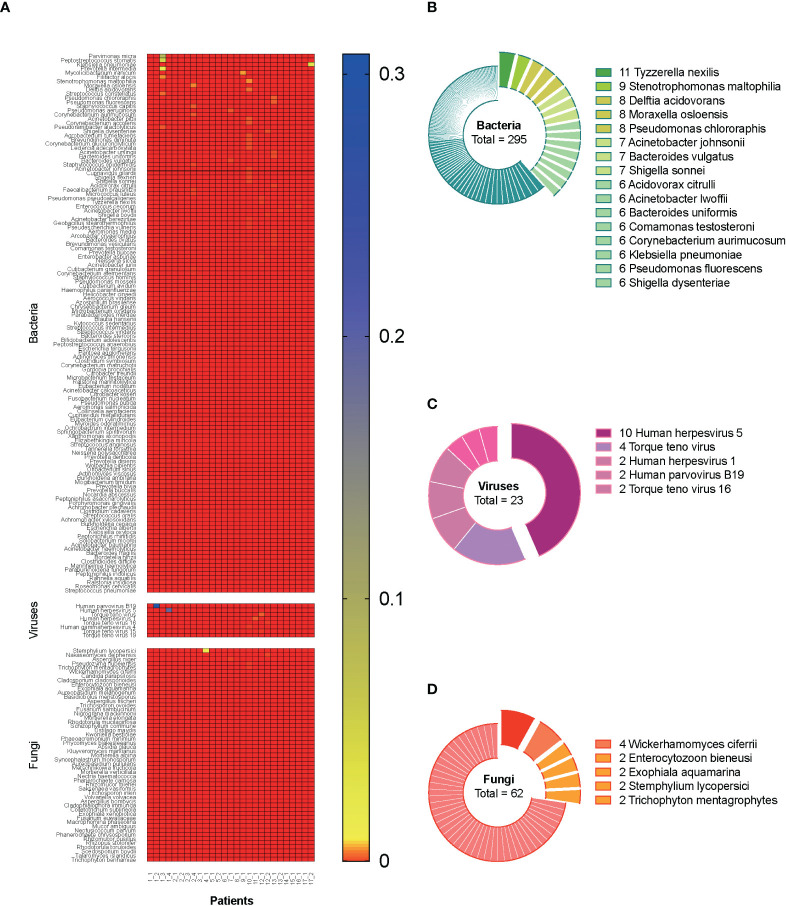
Metagenomic information of pathogens in the plasma. A total of 27 blood samples from 17 included septic CSI patients were tested by mNGS. **(A)** Relative abundance of pathogens in all the blood samples. **(B–D)** The frequency of each identified pathogen. Stratum-specific analyses. N = 27. CSI, cervical spine injury; mNGS, metagenomic next-generation sequencing.

A total of 27 blood samples from 17 included patients were tested by mNGS. **Figure 2A** demonstrates the relative abundance of pathogens in all the blood samples. Overall, 129 bacterial species, 8 viral species, and 51 fungal species were detected by mNGS. Among them, the top 5 abundant bacterial species were *Parvimonas micra*, *Peptostreptococcus stomatis*, *Klebsiella pneumoniae*, *Prevotella intermedia*, and *Mycolicibacterium iranicum*; the top 3 abundant viral species were human parvovirus B19, human herpesvirus 5, and Torque teno virus; and the top 5 abundant fungal species were *Stemphylium lycopersici*, *Nakaseomyces delphensis*, *Aspergillus niger*, *Pseudozyma hubeiensis*, and *Trichophyton mentagrophytes*. We also calculated the frequency of each identified pathogen, as shown in **Figures 2B–D** (stratum-specific analyses). *Tyzzerella nexilis* was most frequently detected by mNGS in plasma of patients with CSI in ICU.

### Comparison of metagenomic next-generation sequencing and conventional culture method

Among the 27 blood samples, blood culture tests were positive only in three (11.1%) of them, while mNGS indicated 23 (85.2%) positive results (reads more than zero). The number of unique reads of pathogens in mNGS-positive samples ranged from three to 5,409. There were two blood samples with both positive blood culture and mNGS results and three with both negative results. Taking blood culture as the gold standard, the sensitivity and specificity of mNGS were 66.7% and 12.5%, respectively, and the positive predictive value (PPV) and negative predictive value (NPV) were 8.7% and 75.0%, respectively (**Table 2**). Combining results of blood culture and other cultures (sputum, catheter drainage fluid, and urine) as the gold standard, sensitivity and specificity of mNGS were 86.4% and 20.0%, respectively (**Table 2**).

**Table 2:** Diagnostic performance of mNGS.

		Blood culture	Combined culture
mNGS (read 0)	Sensitivity (%)	66.7	86.4
	Specificity (%)	12.5	20.0
	PPV (%)	8.7	82.6
	NPV (%)	75.0	25.0
mNGS (read 10)	Sensitivity (%)	33.3	31.8
	Specificity (%)	70.8	80.0
	PPV (%)	12.5	87.5
	NPV (%)	89.5	21.1

mNGS, metagenomic next-generation sequencing; PPV, positive predictive value; NPV, negative predictive value.

**Figure 3:**
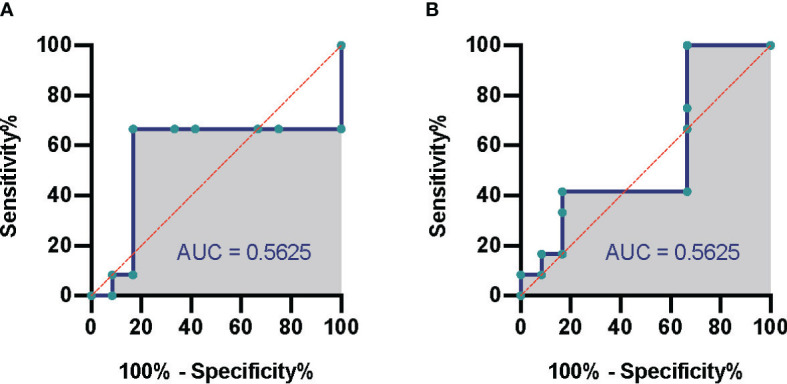
Comparison of mNGS and conventional culture method. The ROC of septic CSI patients under different cultural conditions. **(A)** Blood culture. **(B)** Combined culture. N = 27. mNGS, metagenomic next-generation sequencing; ROC, receiver operating characteristic; CSI, cervical spine injury.

According to the different thresholds of read number to determine the result of mNGS (positive/negative), we also drew the receiver operating characteristic (ROC) curve. **Figures 3A, B** show the data based on blood culture and combined culture, respectively. The area under the curve (AUC) is shown on the graph, which shows low and no significant performance. When the threshold was 10 reads, Youden’s index reached the maximum value. Accordingly, we selected 10 reads as the threshold to distinguish the mNGS results in the subsequent analyses. The sensitivity and specificity of mNGS changed to 33.3% and 70.8% based on the new threshold value (**Table 2**).

### Results of metagenomic next-generation sequencing were not correlated with those of conventional culture

**Figure 4:**
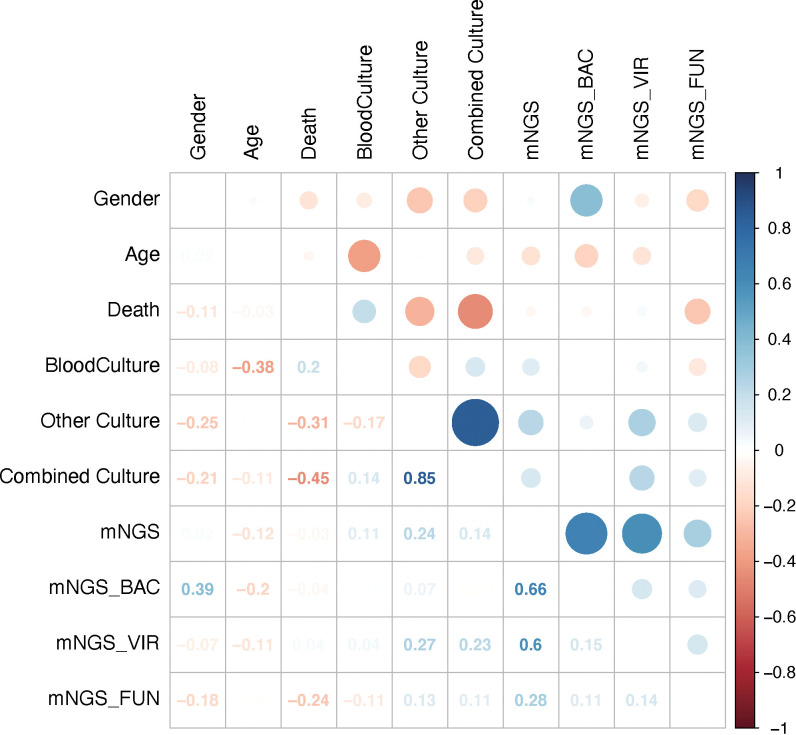
Results of mNGS were not correlated with those of conventional culture. The comparison of the mNGS with different cultured conditions. As shown, mNGS results had little correlation with blood or combined culture results. Correlation analysis. N = 27. mNGS, metagenomic next-generation sequencing.

To explore the relationship between results of mNGS and those of conventional culture, including blood culture and/or other cultures, 10 factors were selected, and correlation analysis was performed. It was out of expectation that mNGS results were not correlated with blood culture or combined culture results (**Figure 4**). Interestingly, results of conventional culture were negatively correlated with death (**Figure 4**), suggesting that positive culture results had an indication of poor prognosis of septic CSI patients.

**Figure 5:**
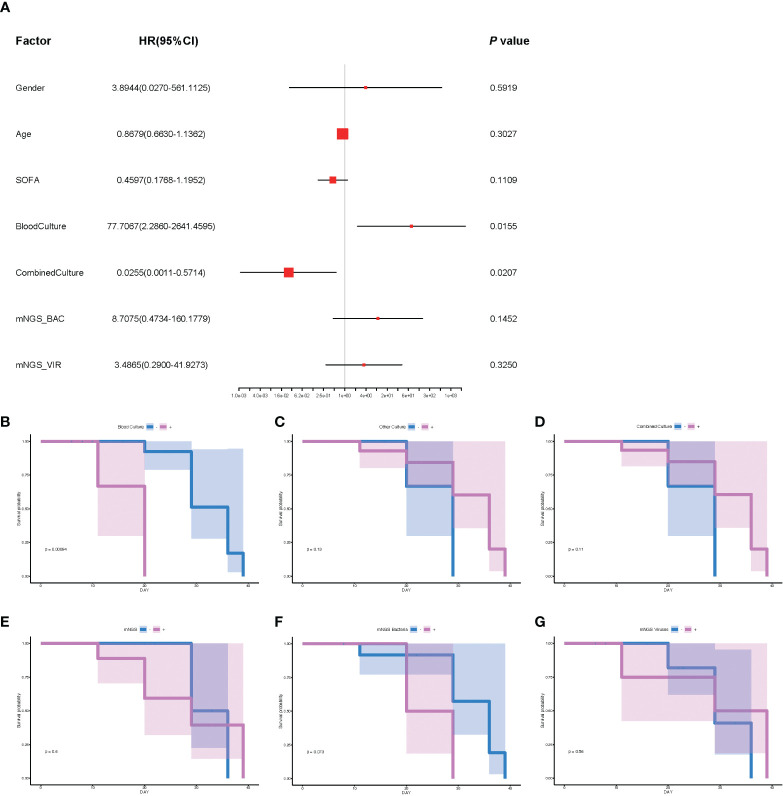
The analysis of prognosis based on the results of mNGS_BAC and mNGS_VIR. **(A)** The correlation of different factors with HR. **(B–G)** Survival probability of different cultural conditions and mNGS data. **(B)** Blood culture. **(C)** Other culture. **(D)** Combine culture. **(E)** mNGS. **(F)** mNGS bacteria. **(G)** mNGS viruses. Cox regression analysis for **(A)** and survival analysis for panels B–G. N = 27. HR, hazard ratio; mNGS, metagenomic next-generation sequencing.

To further explore the underlying factors affecting the prognosis of septic CSI patients in ICU, Cox regression analysis was carried out. As the APACHE II score system was partially coincident with the SOFA score system, only SOFA score on the admission of included patients was selected. Correlation analysis showed that mNGS results were strongly correlated with those of bacteria and viruses, so we chose the results of mNGS-based bacteria (mNGS_BAC) and viruses (mNGS_VIR) to analyze the effects on the prognosis. As shown in **Figure 5A**, the positive result of blood culture was closely correlated with an increased HR (HR 77.7067, 95%CI 2.860–2641.4595, *p* = 0.0155). By contrast, the positive result of combined culture was correlated with a decreased HR (HR 0.0255, 95%CI 0.0011–0.5714, *p* = 0.0207, **Figure 5A**). Because of the fact that there were only three positive results of blood cultures, it was speculated that the septic CSI patients were in the advanced stage when they obtained a positive result of blood culture. In spite of a significant HR of conventional culture being found, neither mNGS_BAC nor mNGS_VIR was shown significantly in the Cox regression analysis, though they appeared to be an increasing trend.

Based on the results of Cox regression analysis, we conducted survival analysis including blood culture and combined culture. The HR for death in the positive results of blood cultures compared with the negative ones was 7.81 (median survival days: 20.0 vs 36.0, *p* = 0.0009, **Figure 5B**). By contrast, survival probability according to other culture, combined culture, mNGS, mNGS_BAC, and mNGS_VIR were not significantly different (**Figures 5C–G**, *p* = 0.13, *p* = 0.11, *p* = 0.6, *p* = 0.073, and *p* = 0.56, respectively).

## Discussion

Sepsis is a life-threatening disease and the main cause of ICU admission and mortality (Engel et al., 2007; Fleischmann et al., 2016; Singer et al., 2016; Cecconi et al., 2018; Weng et al., 2018). CSI accounts for a majority of patients in ICU with a high mortality (Ahuja et al., 2017; Ghajarzadeh et al., 2019). There is increasing evidence that sepsis remains to be one of the major causes of death in patients with CSI in ICU (Cao et al., 2019; Buzzell et al., 2020; Kamp et al., 2020). Thus, timely diagnosis for sepsis and therapeutic actions targeting sepsis is crucial to improving patient prognosis. In this retrospective study, we illustrated the pathogen abundance and frequency map in blood samples of septic patients with CSI in ICU using mNGS and showed a better diagnostic efficacy of mNGS compared with conventional culture in identifying pathogens. In addition, although there was no correlation between mNGS and survival, we found that septic CSI patients in ICU with a positive blood culture had worse outcomes.

Given the fact that mNGS is a potential tool for broad-range pathogen detection and the non-invasive nature of plasma samples, we tried to identify the causative pathogens in the blood of septic CSI patients in ICU using mNGS. A total of 27 blood samples from 17 included patients in our study were tested by mNGS. Metagenomic results showed a wide spectrum of pathogens, including 129 bacterial species, 8 viral species, and 51 fungal species in total. This finding was in agreement with previous case reports and preliminary studies (Abril et al., 2016; Grumaz et al., 2016; Gosiewski et al., 2017; Pan et al., 2017). Although there were no plasma results from other healthy or non-CSI septic patients as controls, this result provided a reference for clinical antibiotic therapy. It should be important to note that, however, microbial nucleic acids have also been reported in healthy volunteers assessed by mNGS (Gosiewski et al., 2017), raising the issue of potential contamination and the question of the clinical significance of DNA detected in plasma.

Previous evidence has suggested different sensitivities and specificities of mNGS for the identification of different types of pathogens (bacterial, viral, or fungal), with sensitivity ranging from 36% to 100% and specificity ranging from 76.5% to 87.5% (Parize et al., 2017; Langelier et al., 2018; Li et al., 2018; Miao et al., 2018). In this study, the sensitivity and specificity of mNGS for pathogens detection were relatively low, possibly due to the lower positivity rate of blood culture and the limited scale of samples. Loose positivity criteria for mNGS, defined as the read number of any identified pathogen more than zero, might also result in lower sensitivity. In addition, we did not find a significant correlation between positive results of mNGS and that of conventional culture. Nevertheless, our result suggested that mNGS was superior to conventional culture in recognizing pathogens in blood, with mNGS indicating 85.2% positive results, while blood culture tests were positive only in 11.1% of all the blood samples, which is compatible with a previous report (Blauwkamp et al., 2019). However, when certain organisms were detected *via* mNGS assay, distinguishing a truly positive sample may require a higher organism concentration to be present. Normalizing the detection threshold to background levels can be helpful to establish sample positivity and avoid false-positive calls. One way to achieve this is to divide the number of sequence reads seen in the sample by the number present in negative or no-template controls and then use a threshold for organism detection (Gu et al., 2019). In this case, we also drew the ROC based on the results of blood culture and combined culture. Unfortunately, no noteworthy performance was found in both models.

In addition to comparing the diagnostic efficiency of mNGS, we also analyzed the effect of positive mNGS consequences on the prognosis of septic patients with CSI in ICU. Several attempts have been made to use metagenomic results as a diagnostic and prognostic marker for sepsis outcomes (Dwivedi et al., 2012; Avriel et al., 2014; Jackson Chornenki et al., 2019). However, in the current study, positive results of mNGS had no prognostic effect on the septic CSI patients in ICU. Positive mNGS results were not correlated with prognosis mainly because this was a retrospective study, and we did not use antibiotics specifically based on the results of mNGS. Further study will be focused on evaluating the effect of mNGS-based therapy on prognosis. By contrast, positive blood culture increases the hazard of death of septic CSI patients in ICU, while positive combined culture result was related to a better prognosis. This abnormal phenomenon can be explained, as the septic CSI patients were in the advanced stage when they obtained a positive result from blood culture. When the infection foci were confined to the primary lesion and the pathogens were not detected in the blood, it was still a relatively early stage of infection for the patients, leading to a better prognosis. It was confirmed by the fact that patients had significantly different survival times according to the results of blood culture, but not to the results of the combined culture or mNGS, which was demonstrated *via* survival analyses in the current study. Taken together, our results suggested practicing vigilance when treating septic patients with CSI in the ICU when the blood culture is positive. Meanwhile, we could use the mNGS assay when the blood culture is negative to improve sensitivity and reduce the risk of death in advance.

Several limitations exist in the current study. First, this study was retrospective and had a small scale of included cases, which led to a bias of conclusions. Second, the aforementioned lack of targeted use of antibiotics based on the results of mNGS led to an inaccurate evaluation of the effect of mNGS on the prognosis. Third, we lacked mNGS results from non-CSI septic patients or non-septic CSI patients as controls.

In conclusion, our study is the first to apply the mNGS of plasma to evaluate the potential impact for septic patients with CSI in ICU, providing evidence for clinicians to use antibiotics when a CSI case is diagnosed with sepsis. While mNGS holds promise for sepsis diagnosis and management, further clinical study is needed to systemically evaluate the value of mNGS in clinical practice.

## Data availability statement

The data presented in the study are deposited in the China National GeneBank DataBase (CNGBdb) repository, accession number CNP0003243.

## Ethics statement

This study was reviewed and approved by The Ethical Review Committee of Changzheng Hospital. The patients/participants provided their written informed consent to participate in this study.

## Author contributions

YS, DY, and ZY conceived and designed the experiments. JW, LD, and QC performed the experiments, analyzed the data, and wrote the manuscript. They contributed equally and were the co-first authors. LW, JB, JH, XL, TZ, and WS performed confirmation experiments. All authors contributed to the article and approved the submitted version.

## Funding

This work is supported by Leading Talent Training in Shanghai Pudong New Area Health System (No. PWRl2018-08), National Natural Science Foundation of China (No. 81872537; 82172183), Project of Clinical Outstanding Clinical Discipline Construction in Shanghai Pudong New Area (No. PWYgy2021-09), Project of Shanghai Municipality Key Medical Specialties Construction (No. ZK2019C08), and Three-year Action Plan for Talent Construction of Changzheng Hospital—Pyramid Talent Project (009).

## Conflict of interest

The authors declare that the research was conducted in the absence of any commercial or financial relationships that could be construed as a potential conflict of interest.

## Publisher’s note

All claims expressed in this article are solely those of the authors and do not necessarily represent those of their affiliated organizations, or those of the publisher, the editors and the reviewers. Any product that may be evaluated in this article, or claim that may be made by its manufacturer, is not guaranteed or endorsed by the publisher.
