# Corrigendum: Potential clinical impact of metagenomic next-generation sequencing of plasma for cervical spine injury with sepsis in ICU: A retrospective study

**DOI:** 10.3389/fcimb.2022.1022341

**Published:** 2022-09-29

**Authors:** Jian Wan, Liwei Duan, Qitong Chen, Lv Wang, Jinxia Bai, Jingyun Hu, Xinyuan Lu, Tao Zhang, Wei Song, Degang Yang, Yi Shan, Zhu Yan

**Affiliations:** ^1^ Department of Emergency and Critical Care Medicine, Shanghai Pudong New Area People’s Hospital, Shanghai, China; ^2^ Department of Emergency and Critical Care Medicine, Shanghai Changzheng Hospital, Shanghai, China; ^3^ Central Lab, Shanghai Key Laboratory of Pathogenic Fungi Medical Testing, Shanghai Pudong New Area People’s Hospital, Shanghai, China; ^4^ Department of Infectious Diseases, Shanghai Skin Disease Hospital, Tongji University School of Medicine, Shanghai, China

**Keywords:** cervical spine injury, metagenomic next-generation sequencing, sepsis, icu, a retrospective study

## Error in author list

In the published article, there was an error in the author list, and author Qitong Chen was erroneously excluded from Equal Authorship. The author list was displayed as “Jian Wan^1†^, Liwei Duan^2†^, Qitong Chen^2^, Lv Wang^2^, Jinxia Bai^1^, Jingyun Hu^3^, Xinyuan Lu^1^, Tao Zhang^1^, Wei Song^1^, Degang Yang^4*^, Yi Shan^2*^, Zhu Yan^2*^ “. The corrected author list appears below.

Jian Wan^1†^, Liwei Duan^2†^, Qitong Chen^2†^, Lv Wang^2^, Jinxia Bai^1^, Jingyun Hu^3^, Xinyuan Lu^1^, Tao Zhang^1^, Wei Song^1^, Degang Yang^4*^, Yi Shan^2*^, Zhu Yan^2*^

†These authors have contributed equally to this work

The authors apologize for this error and state that this does not change the scientific conclusions of the article in any way.

## Publisher’s note

All claims expressed in this article are solely those of the authors and do not necessarily represent those of their affiliated organizations, or those of the publisher, the editors and the reviewers. Any product that may be evaluated in this article, or claim that may be made by its manufacturer, is not guaranteed or endorsed by the publisher.

